# Association Between Exposure to Outdoor Artificial Light at Night and Sleep Disorders Among Children in China

**DOI:** 10.1001/jamanetworkopen.2022.13247

**Published:** 2022-05-20

**Authors:** Le-Bing Wang, Yan-Chen Gong, Qiu-Ling Fang, Xin-Xin Cui, Shyamali C. Dharmage, Bin Jalaludin, Luke D. Knibbs, Michael S. Bloom, Yuming Guo, Li-Zi Lin, Xiao-Wen Zeng, Bo-Yi Yang, Gongbo Chen, Ru-Qing Liu, Yunjiang Yu, Li-Wen Hu, Guang-Hui Dong

**Affiliations:** 1 Guangdong Provincial Engineering Technology Research Center of Environmental Pollution and Health Risk Assessment, Department of Occupational and Environmental Health, School of Public Health, Sun Yat-Sen University, Guangzhou, China; 2 Allergy and Lung Health Unit, Centre for Epidemiology and Biostatistics, School of Population and Global Health, University of Melbourne, Melbourne, Australia; 3 Centre for Research, Evidence Management and Surveillance, South Western Sydney Local Health District, Liverpool, Australia; 4 Ingham Institute for Applied Medical Research, Liverpool, Australia; 5 School of Public Health and Community Medicine Sydney, University of New South Wales, Sydney, Australia; 6 School of Public Health, University of Sydney, Sydney, Australia; 7 Department of Global and Community Health, George Mason University, Fairfax, Virginia; 8 Department of Epidemiology and Preventive Medicine, School of Public Health and Preventive Medicine, Monash University, Melbourne, Australia; 9 State Environmental Protection Key Laboratory of Environmental Pollution Health Risk Assessment, South China Institute of Environmental Sciences, Ministry of Environmental Protection, Guangzhou, China

## Abstract

**Importance:**

Healthy sleep has an important role in the physical and mental health of children. However, few studies have investigated the association between outdoor artificial light at night (ALAN) and sleep disorders in children.

**Objective:**

To explore the associations between outdoor ALAN exposure and sleep disorders in children.

**Design, Setting, and Participants:**

This population-based cross-sectional study, part of the National Chinese Children Health Study, was conducted from April 1, 2012, to June 30, 2013, in the first stage and from May 1, 2016, to May 31, 2018, in the second stage in 55 districts of 14 cities in China. This analysis included 201 994 children and adolescents aged 2 to 18 years. Data were analyzed from February 20 to March 21, 2022.

**Exposures:**

Outdoor ALAN exposure (in nanowatts per centimeters squared per steradian) within 500 m of each participant’s residential address obtained from the satellite imagery data, with a resolution of approximately 500 m.

**Main Outcomes and Measures:**

Sleep disorders were measured by the Chinese version of the Sleep Disturbance Scale for Children. Generalized linear mixed models were used to estimate the associations of outdoor ALAN with sleep scores and sleep disorders.

**Results:**

The study included 201 994 children and adolescents (mean [SD] age, 11.3 [3.2] years; 106 378 boys [52.7%]), 7166 (3.5%) of whom had sleep disorder symptoms. Outdoor ALAN exposure of study participants ranged from 0.02 to 113.48 nW/cm^2^/sr. Compared with the lowest quintile (Q1) of outdoor ALAN exposure, higher quintiles of exposure (Q2-Q5) were associated with an increase in total sleep scores of 0.81 (95% CI, 0.66-0.96) in Q2, 0.83 (95% CI, 0.68-0.97) in Q3, 0.62 (95% CI, 0.46-0.77) in Q4, and 0.53 (95% CI, 0.36-0.70) in Q5. Higher quintiles of exposure were also associated with odds ratios for sleep disorder of 1.34 (95% CI, 1.23-1.45) in Q2, 1.43 (95% CI, 1.32-1.55) in Q3, 1.31 (95% CI, 1.21-1.43) in Q4, and 1.25 (95% CI, 1.14-1.38) in Q5. Similar associations were observed for sleep disorder subtypes. In addition, greater effect estimates were found among children younger than 12 years.

**Conclusions and Relevance:**

The findings of this cross-sectional study suggest that sleep disorders are more prevalent among children residing in areas with high levels of outdoor ALAN and the associations are generally stronger in children younger than 12 years. These findings further suggest that effective control of outdoor ALAN may be an important measure for improving the quality of children’s sleep.

## Key Points

**Question:**

Is artificial light at night (ALAN) associated with sleep disorders in Chinese children?

**Findings:**

In this cross-sectional study including 201 994 participants from the National Chinese Children Health Study, children with high levels of outdoor ALAN exposure had increased sleep scores and increased odds of sleep disorder symptoms. Children’s age modified the associations of outdoor ALAN with sleep score and sleep disorders, and the associations were generally stronger in children younger than 12 years.

**Meaning:**

These findings suggest that reducing the amount of outdoor light pollution in China could reduce the severity of sleep disorders among children.

## Teaser

This cross-sectional study investigates the associations between exposure to outdoor artificial light at night and sleep disorders among a general population of Chinese children.

## Introduction

Healthy sleep is important and critical for physical and mental health in child development.^1,2,3,4^ Approximately 4% of children have a formal diagnosis of a sleep disorder, with large variations in the reported prevalence of different sleep-related problems,^5^ including insufficient sleep quantity and quality, abnormal behavior during sleep, and disturbance of normal rhythmic alterations of sleep and wakefulness.^6^ Circadian rhythm and melatonin secretion are important mechanisms for regulating sleep.^7,8^ Outdoor artificial light at night (ALAN) from sources such as streetlights and billboards^9^ may affect sleep by disturbing the circadian rhythm and inhibiting the secretion of melatonin, leading to sleep disorders.^10,11,12,13,14,15^

During the past few decades, the global proportion of land area exposed to ALAN has increased drastically owing to rapid urbanization. It is estimated that outdoor ALAN increased by 5% to 20% annually in many urban places,^16^ and more than four-fifths of the global population is now affected by nocturnal light pollution.^17^ Previous epidemiological studies in adults suggest that the rapid growth of outdoor ALAN may be associated with a higher risk of poor sleep health.^18,19,20,21,22^ However, few studies have investigated the associations between ALAN exposure and sleep health among children, and the only 2 reports are both from high-income countries.^23,24^ In the first report, a cross-sectional study of 1507 adolescents (aged 9-18 years) from Germany,^23^ participants residing in bright urban areas had a stronger evening-type orientation and later midpoint bedtime than those living in darker rural areas. The second report, a study of 10 123 US adolescents (aged 13-18 years),^24^ found that adolescents residing in the highest quartile of outdoor ALAN had later bedtimes by approximately 30 minutes, and their sleep duration decreased by approximately 11 minutes compared with adolescents residing in the lowest quartile of outdoor ALAN. These 2 studies suggest an association between ALAN exposure and sleep pattern or sleep duration (eg, chronotypes of eveningness and morningness,^23^ bedtime, and sleep length). However, whether ALAN may be associated with other dimensions of sleep disorders in children, such as disorders of initiating and maintaining sleep, remains unclear.

To address these research gaps, we investigated the associations between outdoor ALAN and sleep disorders in a population of Chinese children. We hypothesized that participants with higher outdoor ALAN exposure would have higher sleep scores and greater odds of sleep disturbance in various dimensions.

## Methods

### Study Participants

This cross-sectional study used data from the National Chinese Children Health Study. The steps for selecting the study areas and participants are provided in eFigure 1 in the Supplement. Briefly, we selected Guangdong province in southern China, Liaoning province in northeastern China, and Xinjiang Uygur Autonomous Region in northwestern China, and a 2-stage large epidemiological study was conducted from April 2012 to May 2018. The first stage was conducted from April 1, 2012, to June 30, 2013, in the Seven Northeastern Cities Study. The second stage was conducted from May 1, 2016, to May 31, 2018, in Guangdong and Xinjiang. We randomly selected 55 districts of 14 cities from these 3 regions, including 27 districts in 6 cities in Guangdong (9 districts in Guangzhou, 3 districts in Shenzhen, 5 districts in Foshan, 6 districts in Zhongshan, 2 districts in Zhuhai, and 2 districts in Maoming), 27 districts in 7 cities in Liaoning (6 districts in Shenyang, 5 districts in Dalian, 4 districts in Fushun, 3 districts each in Anshan, Liaoyang, Benxi, and Dandong), and 1 district in Kashgar in Xinjiang Uygur Autonomous Region. Details of all districts are shown in eTable 1 in the Supplement. Finally, we randomly selected 1 or 2 kindergartens, primary schools, and middle schools from the districts of the 14 cities. We invited all children from the selected schools and their parents or guardians to participate in this study. Participating children completed a physical examination and their parents or guardians completed a questionnaire to collect information on sociodemographic factors, environmental exposure, and sleep health. A total of 210 951 children and adolescents aged 2 to 18 years were recruited and 8957 children who did not fully complete the questionnaires were excluded. Finally, 201 994 children were included in this analysis. The study protocol was approved by the Human Studies Committee of Sun Yat-Sen University. All parents or guardians provided written informed consent. We followed the Strengthening the Reporting of Observational Studies in Epidemiology (STROBE) reporting guideline for cross-sectional studies.

### Sleep Disorder Measurements

We used the Chinese version of the Sleep Disturbance Scale for Children (SDSC) to measure each child’s sleep disturbances. The SDSC was first developed and validated among children in Italy and is a suitable tool for assessing sleep disturbances in school-aged children in both clinical and nonclinical settings.^25^ The Chinese version of SDSC has been validated with adequate internal consistency (Cronbach α = 0.81) among children in China.^25,26^ Briefly, the SDSC is a questionnaire that has 26 items related to sleep quality, and the total score is used to evaluate the symptoms of sleep disorder for 6 months.^25^ Two SDSC items are used to assess quality of sleep using a 5-point Likert-type scale for total sleep duration ranging from 1 (9-11 hours) to 5 (<5 hours) and sleep latency ranging from 1 (<15 minutes) to 5 (>60 minutes). To assess frequency of different sleep disorder subtypes, we also used the Likert-type 5-point scale to operationalize the rest of the 24 items as (1) never, (2) occasionally (1-2 times a month), (3) sometimes (1-2 times a week), (4) often (3-5 times a week), and (5) always (6-7 times a week). The 6 types of sleep disorder symptoms included disorders of initiating and maintaining sleep (DIMS), sleep-wake transition disorders, sleep hyperhidrosis (SHY), sleep-breathing disorders (SBD), disorders of arousal, and disorders of excessive somnolence (DOES).^26^ We then calculated the total sleep score and 6 subtype scores for each participant. The possible score ranges of each SDSC scale were 26 to 130 for total sleep score, 7 to 35 for DIMS, 6 to 30 for sleep-wake transition disorders, 5 to 25 for DOES, 3 to 15 for SBD, 3 to 15 for disorders of arousal, and 2 to 10 for SHY. Children with higher scores had a higher risk of sleep disorder than those with low scores. We converted the scores into *t* scores to compare the participant’s total scores and the 6 subtype scores.^26^ Based on the *t* score, children with *t* scores of greater than 70 are considered to have symptoms of a sleep disorder.^25^

We also defined shorter sleep duration (<7 hours) and longer sleep latency (>45 minutes) according to the international consensus recommendations.^4,27^ The items of sleep duration and sleep latency were estimated based on the first 2 items of the SDSC as mentioned earlier.

### Outdoor ALAN Assessment

We downloaded the nighttime light images (ie, monthly cloud-free day-night band composite) produced by Earth Observation Group^28^ from April 2012 to May 2018. The products are composites of cloud-free night images, with stray light filtered out,^29^ derived from the light night images provided by the Visible Infrared Imaging Radiometer Suite day-night band on board the Joint Polar-orbiting Satellite System.^30^

The Visible Infrared Imaging Radiometer Suite day-night band can capture light at wavelengths ranging from 500 to 900 nm by spatial resolution of 15 × 15 arc-second gridded nocturnal luminosity equivalent to 500 × 500 m.^28,29^ The unit of light exposure is expressed as the mean radiance in nanowatts per centimeters squared per steradian (nW/cm^2^/sr).^28,31^ Previous studies have explored the associations between outdoor ALAN through the Visible Infrared Imaging Radiometer Suite day-night band products and health outcomes.^21,32,33,34^ Based on the location of each participant’s residential address and the time of questionnaire survey, we used ArcGIS, version 10.4 (ESRI), to assign the annual mean ALAN exposure within 500 m of each child in the year of questionnaire survey.

### Covariates

We constructed a directed acyclic graph for selecting potential covariates, as shown in eFigure 2 in the Supplement.^35^ Based on the directed acyclic graph and previous literature, we considered the following variables as adjusting covariates: sex (boys or girls),^23,24^ age (in years),^23,24^ parental educational attainment (less than or at least high school),^24^ annual household income (≤¥10 000 [US $1569], ¥10 001-¥30 000 [US $1570-$4708], ¥30 001-¥100 000 [US $4709-$15 694], and >¥100 000 [US $15 695]),^18,24^ district-level gross domestic product (in quartiles),^22,24^ and district-level population density (in quartiles).^23,24,36^ Variables not associated with outdoor ALAN exposure but associated with sleep were used in sensitivity analyses such as passive smoking (yes or no),^37,38^ breastfeeding (yes or no),^39^ asthma (yes or no),^40^ and allergic rhinitis (yes or no).^41,42^ District-level gross domestic product and district-level population density were obtained from the National Bureau of Statistics in 2012 to 2018.^43^ Except for these 2 variables, the covariables mentioned above were all reported by parents or guardians.

### Statistical Analysis

We analyzed data from February 20 to March 21, 2022. We used generalized linear mixed regression models to estimate the associations of outdoor ALAN with sleep scores and sleep disorder. We classified outdoor ALAN in quintiles and used the lowest quintile (Q1) as the reference group. The results are presented as β values (ie, increased *t* scores) or odds ratios (OR, using a logit link in the models) with their corresponding 95% CIs for higher quintiles of exposure (Q2-Q5) compared with Q1 in ALAN. We fitted 2 regression models: (1) a crude model only adjusting for city as a random effect; and (2) a main adjusted model for age, sex, parental educational level, annual household income, district-level gross domestic product, and district-level population density.

We then fitted the corresponding interaction term in adjusted models to investigate potential modifications by child age and sex on the associations of outdoor ALAN with sleep scores and sleep disorders. We dichotomized age at 12 years because children younger than 12 years were mainly in primary school and those 12 years and older were mainly in middle school in China.

We also conducted sensitivity analyses with (1) additional adjusting of the main models for breastfeeding and passive smoking and (2) repeating the analyses excluding children with allergic rhinitis and asthma separately. Statistical analyses were conducted with R software, version 4.0.0 (R Core Team 2020), using a 2-tailed test, with *P* < .05 indicating statistical significance.

## Results

### Study Characteristics

As shown in Table 1, the present study included 201 994 children (response rate, 95.8%). The mean (SD) age was 11.3 (3.2) years, 106 378 (52.7%) were boys, and 95 616 (47.3%) were girls. The prevalence of sleep disorder was 3.5% (7166 participants). Participants in the higher quintiles of exposure were more likely to have a higher parental educational level (eg, 30 723 [76.1%] in Q5 vs 24 416 [60.5%] in Q1), higher annual household income (eg, 7728 [19.1%] in Q5 vs 6048 [15.9%] in Q1), higher prevalence of allergic rhinitis (eg, 6606 [16.4%] in Q4 vs 4536 [11.2%] in Q1), shorter sleep duration (eg, 3751 [9.3%] in Q5 vs 3455 [8.8%] in Q1), and longer sleep latency (eg, 747 [1.9%] in Q5 vs 720 [1.8%] in Q1). The median outdoor ALAN levels were 25.9 (IQR, 17.6-34.8) nW/cm^2^/sr. The detailed distribution of outdoor ALAN levels is presented in eTable 2 in the Supplement and the outdoor ALAN exposure map is presented in the Figure.

**Table 1.** Characteristics of Children Stratified by Outdoor ALAN Exposure

Characteristic	Participant groups^a^					
	All (N = 201 994)	Quintile of outdoor ALAN exposure				
		1 (n = 40 360)	2 (n = 40 455)	3 (n = 40 470)	4 (n = 40 332)	5 (n = 40 377)
Age, mean (SD), y	11.3 (3.2)	11.7 (2.9)	11.4 (3.2)	11.3 (3.1)	11.6 (3.0)	10.6 (3.7)
Sex						
Boys	106 378 (52.7)	21 406 (53.0)	21 308 (52.7)	21 218 (52.4)	21 466 (53.2)	20 900 (51.8)
Girls	95 616 (47.3)	18 954 (47.0)	19 147 (47.3)	19 252 (47.6)	18 866 (46.8)	19 397 (48.1)
Parental educational attainment						
High school or greater	146 944 (72.7)	24 416 (60.5)	29 300 (72.4)	31 448 (77.7)	31 057 (77.0)	30 723 (76.2)
Less than high school	55 050 (27.3)	15 944 (39.5)	11 155 (27.6)	9022 (22.3)	9275 (23.0)	9654 (23.9)
Annual household income, ¥ (US$)						
≤10 000 (1569)	43 101 (21.3)	10 924 (27.1)	8663 (21.4)	8065 (19.9)	8112 (20.1)	7337 (18.2)
10 001-30 000 (1570-4708)	46 867 (23.2)	10 503 (26.0)	9218 (22.8)	9579 (23.7)	7885 (19.5)	9682 (24.0)
30 001-100 000 (4709-15 694)	68 534 (33.9)	12 525 (31.0)	13 728 (33.9)	13 895 (34.3)	12 756 (31.6)	15 630 (38.7)
>100 000 (15 695)	43 492 (21.5)	6408 (15.9)	8846 (21.9)	8931 (22.1)	11 579 (28.7)	7728 (19.1)
Breastfeeding						
No	49 304 (24.4)	8871 (22.0)	9772 (24.1)	10 115 (25.0)	9159 (22.7)	11 387 (28.2)
Yes	152 690 (75.6)	31 489 (78.0)	30 683 (75.8)	30 355 (75.0)	31 173 (77.3)	28 990 (71.9)
Passive smoking						
No	122 162 (60.5)	23 679 (58.7)	24 767 (61.2)	24 511 (60.6)	25 056 (62.1)	24 149 (59.9)
Yes	79 832 (39.5)	16 681 (41.3)	15 688 (38.8)	15 959 (39.4)	15 276 (37.9)	16 228 (40.2)
Asthma						
No	185 260 (91.7)	36 918 (91.5)	36 743 (90.8)	37 311 (92.2)	37 028 (91.8)	37 260 (92.4)
Yes	16 734 (8.3)	3442 (8.5)	3712 (9.2)	3159 (7.8)	3304 (8.2)	3117 (7.7)
Allergic rhinitis						
No	175 539 (86.9)	35 824 (88.8)	35 136 (86.9)	34 708 (85.8)	33 726 (83.6)	36 145 (89.6)
Yes	26 455 (13.1)	4536 (11.2)	5319 (13.1)	5762 (14.2)	6606 (16.4)	4232 (10.5)
District-level gross domestic product, ¥ (US$) per capita^b^						
≤51 042 (8011)	53 779 (26.6)	10 734 (26.6)	12 506 (30.9)	13 000 (32.1)	9335 (23.1)	8204 (20.3)
51 900-99 754 (8146-15 656)	48 080 (23.8)	10 906 (27.0)	6790 (16.8)	9989 (24.7)	7259 (18.0)	13 136 (32.6)
101 141-125 585 (15 874-19 710)	49 905 (24.7)	7330 (18.2)	15 415 (38.1)	7328 (18.1)	7118 (17.6)	12 714 (31.5)
141 553-297 179 (22 216-46 641)	50 230 (24.9)	11 390 (28.2)	5744 (14.2)	10 153 (25.1)	16 620 (41.2)	6323 (15.7)
District-level population density, persons/km^2^^b^						
≤971	59 511 (29.5)	22 771 (56.4)	10 823 (26.7)	9346 (23.1)	8903 (22.1)	7668 (19.0)
1014-2338	42 837 (21.2)	9569 (23.7)	13 540 (33.5)	11 938 (29.5)	6162 (15.3)	1628 (4.0)
2358-7940	55 843 (27.6)	3717 (9.2)	13 463 (33.3)	11 785 (29.1)	12 840 (31.8)	14 038 (34.8)
8325-34 432	43 803 (21.7)	4303 (10.7)	2629 (6.5)	7401 (18.3)	12 427 (30.8)	17 043 (42.3)
Sleep disorder	7166 (3.5)	1230 (3.0)	1480 (3.7)	1568 (3.9)	1532 (3.8)	1356 (3.4)
SWTD	8698 (4.3)	1621 (4.0)	1767 (4.4)	1867 (4.6)	1705 (4.2)	1738 (4.3)
DIMS	9941 (4.9)	1796 (4.4)	2098 (5.2)	2125 (5.3)	2169 (5.4)	1753 (4.3)
DOES	9760 (4.8)	1631 (4.0)	2006 (5.0)	2012 (5.0)	2037 (5.1)	2074 (5.1)
DA	12 100 (6.0)	2421 (6.0)	2392 (5.9)	2341 (5.8)	2354 (5.8)	2592 (6.4)
SHY	14 053 (7.0)	2752 (6.8)	2949 (7.3)	2951 (7.3)	2808 (7.0)	2593 (6.4)
SBD	10 398 (5.1)	1691 (4.2)	2032 (5.0)	2188 (5.4)	2238 (5.5)	2249 (5.6)
Shorter sleep duration	20 638 (10.2)	3544 (8.8)	4573 (11.3)	3939 (9.7)	4831 (12.0)	3751 (9.3)
Longer sleep latency	3727 (1.8)	720 (1.8)	796 (2.0)	737 (1.8)	727 (1.8)	747 (1.9)
ALAN, median (IQR), nW/cm^2^/sr	25.9 (17.6-34.8)	8.5 (4.6-11.6)	19.1 (17.6-20.8)	25.9 (24.4-27.3)	32.4 (30.6-34.8)	47.7 (42.0-64.0)

Abbreviations: ALAN, artificial light at night; DA, disorders of arousal; DIMS, disorders of initiating and maintaining sleep; DOES, disorders of excessive somnolence; nW/cm^2^/sr, nanowatts per centimeters squared per steradian; SHY, sleep hyperhidrosis; SBD, sleep-breathing disorders; SWTD, sleep-wake transition disorders.

^a^ Unless otherwise indicated, data are expressed as number (%) of participants. Percentages have been rounded and may not total 100.

^b^ Categories are ordered in quartiles.

**Figure.**
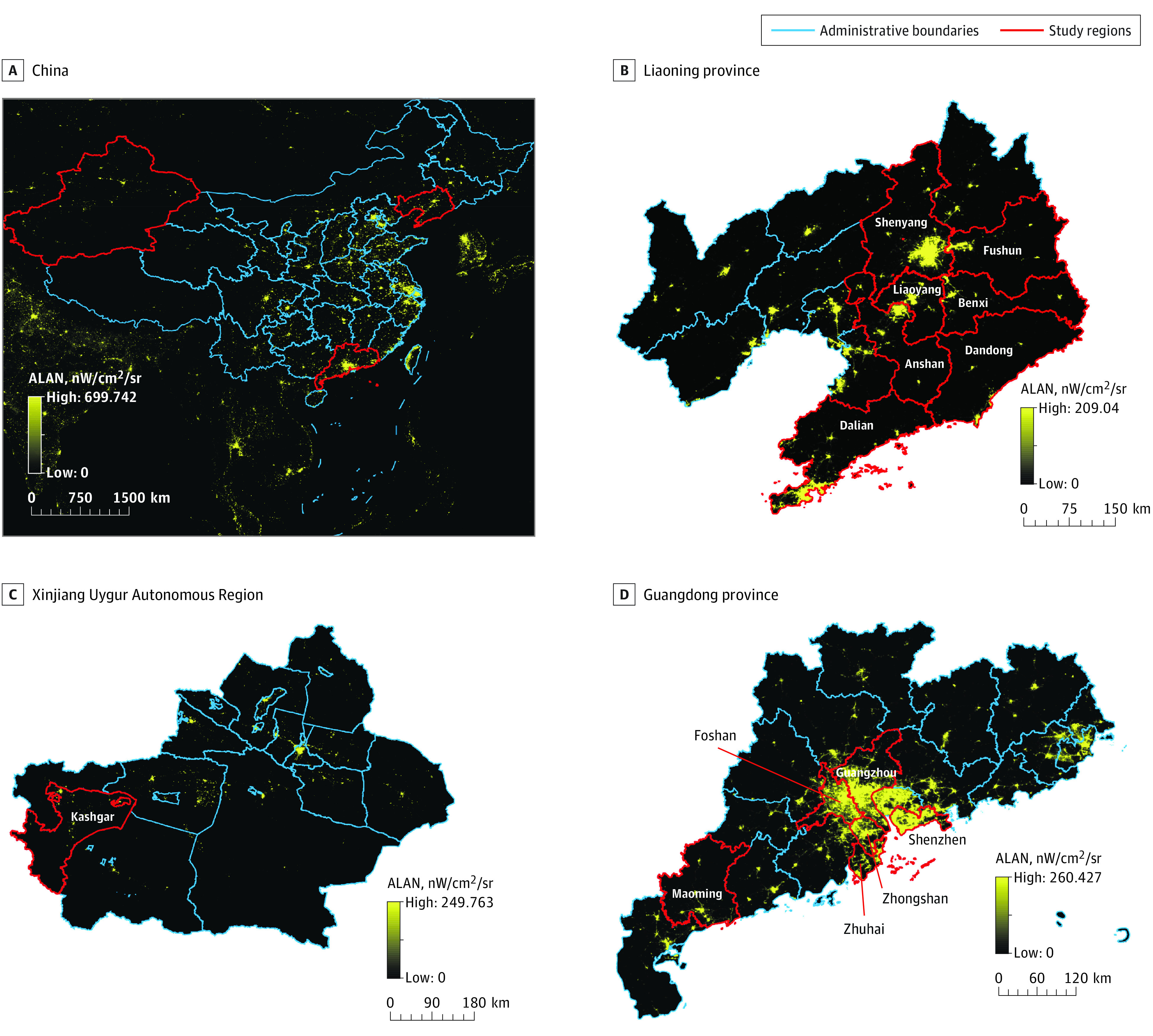
Maps of Artificial Light at Night (ALAN) Exposure in China in 2016 Artificial light at night was measured as nanowatts per centimeters squared per steradian (nW/cm^2^/sr).

### Associations Between Quintiles of Outdoor ALAN Exposure and Sleep Disorder

Table 2 shows the associations of outdoor ALAN exposure with sleep score and sleep disorder. In an adjusted model, outdoor ALAN was associated with increased total sleep scores compared with Q1 (Q2, β = 0.81 [95% CI, 0.66-0.96]; Q3, β = 0.83 [95% CI, 0.68-0.97]; Q4, β = 0.62 [95% CI, 0.46-0.77]; and Q5, β = 0.53 [95% CI, 0.36-0.70]). The associations between outdoor ALAN exposure and increased subtype scores were similar. Compared with Q1 of outdoor ALAN, the ORs of sleep disorders were higher among those in higher quintiles of exposure (Q2, 1.34 [95% CI, 1.23-1.45]; Q3, 1.43 [95% CI, 1.32-1.55]; Q4, 1.31 [95% CI, 1.21-1.43]; and Q5, 1.25 [95% CI, 1.14-1.38]). The outdoor ALAN exposure was also positively associated with sleep disorder subtypes except for disorders of arousal, for which ORs ranged from 1.07 (95% CI,1.01-1.14) for SHY to 1.35 (95% CI, 1.24-1.47) for DOES. Compared with Q1 of outdoor ALAN, Q5 exposure was also associated with an OR of 1.37 (95% CI, 1.28-1.47) for shorter sleep duration and 1.33 (95% CI, 1.17-1.51) for longer sleep latency.

**Table 2.** Associations Between Quintiles of Outdoor ALAN Exposure With Sleep Disorder Score and Odds of Sleep Disorder in Children

Variable	Quintile of outdoor ALAN exposure^a^				
	1	2	3	4	5
**Sleep disorder scores**					
Total sleep, β (95% CI)^b^					
Crude	1 [Reference]	0.83 (0.69 to 0.98)	0.88 (0.74 to 1.02)	0.74 (0.60 to 0.88)	0.69 (0.53 to 0.84)
Adjusted^c^	1 [Reference]	0.81 (0.66 to 0.96)	0.83 (0.68 to 0.97)	0.62 (0.46 to 0.77)	0.53 (0.36 to 0.70)
DIMS, β (95% CI)^b^					
Crude	1 [Reference]	0.97 (0.83 to 1.11)	0.90 (0.77 to 1.04)	0.91 (0.77 to 1.05)	0.96 (0.81 to 1.12)
Adjusted^c^	1 [Reference]	0.90 (0.75 to 1.04)	0.76 (0.61 to 0.90)	0.74 (0.59 to 0.89)	0.85 (0.68 to 1.02)
SBD, β (95% CI)^b^					
Crude	1 [Reference]	0.56 (0.42 to 0.70)	0.86 (0.72 to 1.00)	0.89 (0.75 to 1.03)	0.84 (0.68 to 1.00)
Adjusted^c^	1 [Reference]	0.41 (0.27 to 0.56)	0.62 (0.48 to 0.77)	0.53 (0.37 to 0.68)	0.35 (0.17 to 0.52)
DA, β (95% CI)^b^					
Crude	1 [Reference]	0.16 (0.02 to 0.30)	0.06 (−0.08 to 0.20)	0.01 (−0.13 to 0.15)	−0.08 (−0.23 to 0.08)
Adjusted^c^	1 [Reference]	0.28 (0.13 to 0.42)	0.24 (0.09 to 0.39)	0.17 (0.02 to 0.32)	0.10 (−0.07 to 0.27)
SWTD, β (95% CI)^b^					
Crude	1 [Reference]	0.47 (0.33 to 0.61)	0.41 (0.27 to 0.55)	0.20 (0.06 to 0.34)	0.16 (0.00 to 0.32)
Adjusted^c^	1 [Reference]	0.40 (0.25 to 0.54)	0.41 (0.26 to 0.55)	0.16 (0.01 to 0.32)	−0.03 (−0.20 to 0.15)
DOES, β (95% CI)^b^					
Crude	1 [Reference]	0.48 (0.34 to 0.63)	0.68 (0.54 to 0.82)	0.57 (0.43 to 0.72)	0.47 (0.31 to 0.63)
Adjusted^c^	1 [Reference]	0.68 (0.54 to 0.83)	0.77 (0.62 to 0.92)	0.57 (0.42 to 0.73)	0.67 (0.50 to 0.84)
SHY, β (95% CI)^b^					
Crude	1 [Reference]	0.62 (0.47 to 0.76)	0.55 (0.41 to 0.69)	0.35 (0.21 to 0.49)	0.32 (0.16 to 0.48)
Adjusted^c^	1 [Reference]	0.42 (0.27 to 0.56)	0.47 (0.32 to 0.61)	0.25 (0.10 to 0.40)	−0.04 (−0.21 to 0.13)
**Odds of sleep disorder**					
Sleep disorder, OR (95% CI)					
Crude	1 [Reference]	1.23 (1.14 to 1.33)	1.30 (1.21 to 1.41)	1.22 (1.13 to 1.32)	1.14 (1.05 to 1.25)
Adjusted^c^	1 [Reference]	1.34 (1.23 to 1.45)	1.43 (1.32 to 1.55)	1.31 (1.21 to 1.43)	1.25 (1.14 to 1.38)
DIMS, OR (95% CI)					
Crude	1 [Reference]	1.26 (1.18 to 1.35)	1.29 (1.21 to 1.38)	1.26 (1.18 to 1.35)	1.25 (1.16 to 1.35)
Adjusted^c^	1 [Reference]	1.29 (1.21 to 1.38)	1.32 (1.23 to 1.41)	1.28 (1.19 to 1.37)	1.31 (1.20 to 1.43)
SBD, OR (95% CI)					
Crude	1 [Reference]	1.17 (1.09 to 1.25)	1.26 (1.18 to 1.35)	1.29 (1.21 to 1.38)	1.25 (1.16 to 1.34)
Adjusted^c^	1 [Reference]	1.17 (1.09 to 1.25)	1.23 (1.15 to 1.32)	1.21 (1.12 to 1.30)	1.15 (1.06 to 1.24)
DA, OR (95% CI)					
Crude	1 [Reference]	0.97 (0.91 to 1.03)	0.93 (0.87 to 0.98)	0.93 (0.88 to 0.99)	0.94 (0.88 to 1.01)
Adjusted^c^	1 [Reference]	1.04 (0.98 to 1.11)	1.03 (0.96 to 1.09)	1.03 (0.97 to 1.10)	1.07 (0.99 to 1.15)
SWTD, OR (95% CI)					
Crude	1 [Reference]	1.10 (1.03 to 1.18)	1.12 (1.05 to 1.20)	1.04 (0.97 to 1.12)	1.00 (0.93 to 1.08)
Adjusted^c^	1 [Reference]	1.16 (1.08 to 1.25)	1.21 (1.12 to 1.30)	1.11 (1.02 to 1.19)	1.05 (0.96 to 1.14)
DOES, OR (95% CI)					
Crude	1 [Reference]	1.20 (1.12 to 1.28)	1.23 (1.15 to 1.32)	1.19 (1.11 to 1.27)	1.19 (1.10 to 1.28)
Adjusted^c^	1 [Reference]	1.31 (1.22 to 1.41)	1.33 (1.24 to 1.43)	1.27 (1.18 to 1.37)	1.35 (1.24 to 1.47)
SHY, OR (95% CI)					
Crude	1 [Reference]	1.15 (1.09 to 1.21)	1.09 (1.04 to 1.16)	1.06 (1.00 to 1.12)	1.02 (0.96 to 1.09)
Adjusted^c^	1 [Reference]	1.11 (1.05 to 1.18)	1.11 (1.05 to 1.18)	1.07 (1.01 to 1.14)	0.94 (0.87 to 1.01)
Shorter sleep duration, OR (95% CI)					
Crude	1 [Reference]	1.33 (1.27 to 1.40)	1.33 (1.27 to 1.40)	1.17 (1.12 to 1.23)	1.51 (1.43 to 1.60)
Adjusted^c^	1 [Reference]	1.23 (1.17 to 1.30)	1.26 (1.19 to 1.34)	1.14 (1.08 to 1.21)	1.37 (1.28 to 1.47)
Longer sleep latency, OR (95% CI)					
Crude	1 [Reference]	1.19 (1.08 to 1.32)	1.09 (0.98 to 1.21)	1.08 (0.97 to 1.20)	1.25 (1.11 to 1.41)
Adjusted^c^	1 [Reference]	1.22 (1.09 to 1.35)	1.10 (0.99 to 1.23)	1.09 (0.97 to 1.22)	1.33 (1.17 to 1.51)

Abbreviations: ALAN, artificial light at night; DA, disorders of arousal; DIMS, disorders of initiating and maintaining sleep; DOES, disorders of excessive somnolence; OR, odds ratio; SBD, sleep-breathing disorders; SHY, sleep hyperhidrosis; SWTD, sleep-wake transition disorders.

^a^ Includes 201 994 participants. Median ALAN exposure was 8.5 (IQR, 4.6-11.6) nanowatts/centimeters squared/steradian (nW/cm^2^/sr) for quintile 1; 19.1 (IQR, 17.6-20.8) nW/cm^2^/sr for quintile 2; 25.9 (IQR, 24.4-27.3) nW/cm^2^/sr for quintile 3; 32.4 (IQR, 30.6-34.8) nW/cm^2^/sr for quintile 4; and 47.7 (IQR, 42.0-64.0) nW/cm^2^/sr for quintile 5.

^b^ Scores were calculated as *t* scores.

^c^ Adjusted for age, sex, parental educational attainment, annual household income, district-level gross domestic product, and district-level population density.

### Potential Modification

Child age significantly modified the associations of outdoor ALAN exposure with subtypes score, such as the DIMS, SBD, sleep-wake transition disorder, DOES, and SHY scores. The associations were stronger in children younger than 12 years except for DOES score (Table 3). With the DIMS score, for example, compared with Q1 of outdoor ALAN exposure, higher quintiles of exposure were associated with increases in scores of 1.25 (95% CI, 1.05-1.45) in Q2, 1.19 (95% CI, 0.99-1.39) in Q3, 0.99 (95% CI, 0.78-1.20) in Q4, and 1.31 (95% CI, 1.09-1.53) in Q5 among children younger than 12 years, but the increases were only 0.55 (95% CI, 0.35- 0.76) in Q2, 0.35 (95% CI, 0.14-0.55) in Q3, 0.51 (95% CI, 0.30-0.71) in Q4, and 0.17 (95% CI, −0.06 to 0.39) in Q5 among children 12 years or older (*P* < .001 for interaction). Similarly, the ORs for the subtypes of sleep disorder were also higher in children younger than 12 years, except in DOES and shorter sleep duration. For example, compared with Q1 of outdoor ALAN exposure, higher quintiles of exposure were associated with ORs for DIMS of 1.47 (95% CI, 1.33-1.63) in Q2, 1.46 (95% CI, 1.32-1.61) in Q3, 1.39 (95% CI, 1.26-1.54) in Q4, 1.49 (95% CI, 1.34-1.66) in Q5 among children younger than 12 years, but the ORs were only 1.16 (95% CI, 1.06-1.27) in Q2, 1.23 (95% CI, 1.12-1.35) in Q3, 1.20 (95% CI, 1.09-1.31) in Q4, 1.14 (95% CI, 1.03-1.27) in Q5 among children 12 years or older (*P* = .001 for interaction) (Table 3). Child sex only modified the association between outdoor ALAN exposure and SBD score (Table 4), and the association was generally stronger among boys than among girls (eg, β = 0.69 [95% CI, 0.49-0.89] vs β = 0.55 [95% CI, 0.34-0.75] in Q3; β = 0.70 [95% CI, 0.50-0.90] vs β = 0.33 [95% CI, 0.12-0.55] in Q4; β = 0.43 [95% CI, 0.21-0.65] vs β = 0.25 [95% CI, 0.03-0.47] in Q5; *P*  =  .02 for interaction). However, no modification of sex was found in the associations of ALAN and the other subtypes of sleep disorders.

**Table 3.** Associations of Quintiles of Outdoor ALAN Exposure With Sleep Disorder Score and Odds of Sleep Disorder in Children by Age

Variable by age, y	Quintile of outdoor ALAN exposure^a^					*P* value for interaction^b^
	1	2	3	4	5	
Sleep disorder score						
Total sleep, β (95% CI)^c^						
<12	1 [Reference]	0.93 (0.73 to 1.13)	1.00 (0.80 to 1.20)	0.75 (0.54 to 0.96)	0.73 (0.51 to 0.95)	.05
≥12	1 [Reference]	0.68 (0.48 to 0.89)	0.63 (0.42 to 0.84)	0.47 (0.26 to 0.68)	0.35 (0.12 to 0.58)	
DIMS, β (95% CI)^c^						
<12	1 [Reference]	1.25 (1.05 to 1.45)	1.19 (0.99 to 1.39)	0.99 (0.78 to 1.20)	1.31 (1.09 to 1.53)	<.001
≥12	1 [Reference]	0.55 (0.35 to 0.76)	0.35 (0.14 to 0.55)	0.51 (0.30 to 0.71)	0.17 (−0.06 to 0.39)	
SBD, β (95% CI)^c^						
<12	1 [Reference]	0.53 (0.33 to 0.73)	0.91 (0.71 to 1.11)	0.76 (0.55 to 0.97)	0.90 (0.68 to 1.12)	<.001
≥12	1 [Reference]	0.29 (0.09 to 0.50)	0.29 (0.08 to 0.49)	0.26 (0.05 to 0.47)	−0.13 (−0.36 to 0.09)	
DA, β (95% CI)^c^						
<12	1 [Reference]	0.35 (0.15 to 0.55)	0.15 (−0.05 to 0.35)	0.17 (−0.04 to 0.38)	−0.15 (−0.37 to 0.07)	<.001
≥12	1 [Reference]	0.19 (−0.01 to 0.40)	0.33 (0.12 to 0.54)	0.17 (−0.04 to 0.37)	0.47 (0.24 to 0.70)	
SWTD, β (95% CI)^c^						
<12	1 [Reference]	0.52 (0.32 to 0.72)	0.60 (0.40 to 0.80)	0.34 (0.13 to 0.55)	0.56 (0.34 to 0.78)	<.001
≥12	1 [Reference]	0.27 (0.06 to 0.47)	0.14 (−0.07 to 0.35)	−0.07 (−0.28 to 0.14)	−0.43 (−0.65 to −0.20)	
DOES, β (95% CI)^c^						
<12	1 [Reference]	0.38 (0.18 to 0.58)	0.55 (0.35 to 0.75)	0.40 (0.19 to 0.61)	−0.22 (−0.44 to 0.00)	<.001
≥12	1 [Reference]	1.01 (0.80 to 1.21)	1.08 (0.87 to 1.28)	0.83 (0.62 to 1.03)	1.36 (1.14 to 1.59)	
SHY, β (95% CI)^c^						
<12	1 [Reference]	0.62 (0.42 to 0.82)	0.49 (0.29 to 0.69)	0.34 (0.14 to 0.54)	0.44 (0.23 to 0.65)	.002
≥12	1 [Reference]	0.17 (−0.03 to 0.37)	0.30 (0.10 to 0.51)	0.05 (−0.16 to 0.25)	−0.06 (−0.28 to 0.17)	
**Odds of sleep disorder **						
Sleep disorder, OR (95% CI)						
<12	1 [Reference]	1.27 (1.14 to 1.43)	1.34 (1.19 to 1.50)	1.30 (1.16 to 1.46)	1.18 (1.04 to 1.33)	.35
≥12	1 [Reference]	1.41 (1.26 to 1.57)	1.54 (1.38 to 1.72)	1.34 (1.20 to 1.50)	1.31 (1.16 to 1.49)	
DIMS, OR (95% CI)						
<12	1 [Reference]	1.47 (1.33 to 1.63)	1.46 (1.32 to 1.61)	1.39 (1.26 to 1.54)	1.49 (1.34 to 1.66)	.001
≥12	1 [Reference]	1.16 (1.06 to 1.27)	1.23 (1.12 to 1.35)	1.20 (1.09 to 1.31)	1.14 (1.03 to 1.27)	
SBD, OR (95% CI)						
<12	1 [Reference]	1.12 (1.02 to 1.23)	1.26 (1.15 to 1.39)	1.25 (1.13 to 1.37)	1.23 (1.12 to 1.36)	.007
≥12	1 [Reference]	1.23 (1.11 to 1.36)	1.19 (1.07 to 1.32)	1.15 (1.04 to 1.28)	1.07 (0.95 to 1.19)	
DA, OR (95% CI)						
<12	1 [Reference]	1.03 (0.95 to 1.12)	0.95 (0.87 to 1.04)	1.00 (0.92 to 1.10)	0.95 (0.87 to 1.04)	<.001
≥12	1 [Reference]	1.05 (0.96 to 1.14)	1.12 (1.02 to 1.22)	1.06 (0.97 to 1.16)	1.22 (1.11 to 1.34)	
SWTD, OR (95% CI)						
<12	1 [Reference]	1.12 (1.02 to 1.23)	1.12 (1.01 to 1.23)	1.04 (0.94 to 1.15)	1.02 (0.92 to 1.14)	.21
≥12	1 [Reference]	1.22 (1.09 to 1.35)	1.31 (1.18 to 1.46)	1.18 (1.06 to 1.32)	1.10 (0.98 to 1.24)	
DOES, OR (95% CI)						
<12	1 [Reference]	1.17 (1.05 to 1.31)	1.21 (1.08 to 1.35)	1.20 (1.07 to 1.35)	1.04 (0.93 to 1.18)	<.001
≥12	1 [Reference]	1.42 (1.30 to 1.55)	1.44 (1.32 to 1.58)	1.34 (1.23 to 1.47)	1.50 (1.36 to 1.66)	
SHY, OR (95% CI)						
<12	1 [Reference]	1.13 (1.06 to 1.21)	1.06 (0.99 to 1.14)	1.08 (1.00 to 1.16)	1.06 (0.98 to 1.14)	.01
≥12	1 [Reference]	1.09 (0.99 to 1.21)	1.17 (1.06 to 1.3)	1.03 (0.93 to 1.15)	0.93 (0.83 to 1.04)	
Shorter sleep duration, OR (95% CI)						
<12	1 [Reference]	1.16 (1.03 to 1.30)	0.94 (0.83 to 1.06)	0.80 (0.71 to 0.90)	0.62 (0.54 to 0.72)	<.001
≥12	1 [Reference]	1.33 (1.26 to 1.41)	1.39 (1.31 to 1.48)	1.28 (1.21 to 1.36)	1.57 (1.47 to 1.68)	
Longer sleep latency, OR (95% CI)						
<12	1 [Reference]	1.50 (1.27 to 1.78)	1.31 (1.11 to 1.56)	1.18 (0.98 to 1.41)	1.88 (1.58 to 2.23)	<.001
≥12	1 [Reference]	1.07 (0.93 to 1.22)	1.00 (0.87 to 1.15)	1.03 (0.90 to 1.18)	0.94 (0.80 to 1.11)	

Abbreviations: ALAN, artificial light at night; DA, disorders of arousal; DIMS, disorders of initiating and maintaining sleep; DOES, disorders of excessive somnolence; OR, odds ratio; SBD, sleep-breathing disorders; SHY, sleep hyperhidrosis; SWTD, sleep-wake transition disorders.

^a^ Includes 201 994 participants. Median ALAN exposure was 8.5 (IQR, 4.6-11.6) nanowatts/centimeters squared/steradian (nW/cm^2^/sr) for quintile 1; 19.1 (IQR, 17.6-20.8) nW/cm^2^/sr for quintile 2; 25.9 (IQR, 24.4-27.3) nW/cm^2^/sr for quintile 3; 32.4 (IQR, 30.6-34.8) nW/cm^2^/sr for quintile 4; and 47.7 (IQR, 42.0-64.0) nW/cm^2^/sr for quintile 5. Values are adjusted for sex, parental educational attainment, annual household income, district-level gross domestic product, district-level population density, and interaction item (ALAN exposure × age).

^b^ Calculated by adding an interaction item in the model.

^c^ Scores were calculated as *t* scores.

**Table 4.** Associations of Quintiles of Outdoor ALAN Exposure With Sleep Disorder Score and Odds of Sleep Disorder in Children by Sex (N = 201 994)^a^

Variable by sex	Quintile of outdoor ALAN exposure^a^					*P* value for interaction^b^
	1	2	3	4	5	
**Sleep disorder score**						
Total sleep, β (95% CI)^c^						
Boys	1 [Reference]	0.80 (0.60 to 1.00)	0.91 (0.71 to 1.11)	0.78 (0.58 to 0.98)	0.60 (0.38 to 0.82)	.05
Girls	1 [Reference]	0.82 (0.62 to 1.03)	0.73 (0.52 to 0.93)	0.43 (0.22 to 0.65)	0.45 (0.23 to 0.68)	
DIMS, β (95% CI)^c^						
Boys	1 [Reference]	0.94 (0.75 to 1.13)	0.91 (0.71 to 1.11)	0.86 (0.66 to 1.06)	0.95 (0.73 to 1.17)	.14
Girls	1 [Reference]	0.85 (0.64 to 1.05)	0.59 (0.39 to 0.80)	0.61 (0.39 to 0.82)	0.73 (0.51 to 0.96)	
SBD, β (95% CI)^c^						
Boys	1 [Reference]	0.38 (0.19 to 0.57)	0.69 (0.49 to 0.89)	0.70 (0.50 to 0.90)	0.43 (0.21 to 0.65)	.02
Girls	1 [Reference]	0.45 (0.24 to 0.65)	0.55 (0.34 to 0.75)	0.33 (0.12 to 0.55)	0.25 (0.03 to 0.47)	
DA, β (95% CI)^c^						
Boys	1 [Reference]	0.31 (0.11 to 0.51)	0.27 (0.07 to 0.47)	0.29 (0.09 to 0.49)	0.12 (−0.10 to 0.34)	.45
Girls	1 [Reference]	0.24 (0.03 to 0.45)	0.20 (−0.01 to 0.41)	0.04 (−0.18 to 0.25)	0.08 (−0.15 to 0.30)	
SWTD, β (95% CI)^c^						
Boys	1 [Reference]	0.36 (0.16 to 0.56)	0.42 (0.22 to 0.62)	0.25 (0.05 to 0.45)	−0.03 (−0.25 to 0.19)	.39
Girls	1 [Reference]	0.44 (0.23 to 0.64)	0.39 (0.18 to 0.60)	0.06 (−0.15 to 0.27)	−0.02 (−0.25 to 0.20)	
DOES, β (95% CI)^c^						
Boys	1 [Reference]	0.63 (0.44 to 0.82)	0.78 (0.58 to 0.98)	0.66 (0.46 to 0.86)	0.70 (0.49 to 0.91)	.25
Girls	1 [Reference]	0.75 (0.55 to 0.96)	0.76 (0.55 to 0.96)	0.47 (0.26 to 0.68)	0.63 (0.41 to 0.86)	
SHY, β (95% CI)^c^						
Boys	1 [Reference]	0.44 (0.25 to 0.63)	0.57 (0.38 to 0.76)	0.41 (0.21 to 0.61)	0.03 (−0.18 to 0.24)	.11
Girls	1 [Reference]	0.39 (0.19 to 0.59)	0.35 (0.15 to 0.55)	0.07 (−0.13 to 0.28)	−0.11 (−0.33 to 0.10)	
**Odds of sleep disorder**						
Sleep disorder, OR (95% CI)						
Boys	1 [Reference]	1.31 (1.18 to 1.46)	1.42 (1.27 to 1.58)	1.36 (1.21 to 1.51)	1.24 (1.09 to 1.39)	.61
Girls	1 [Reference]	1.37 (1.22 to 1.53)	1.45 (1.29 to 1.63)	1.27 (1.12 to 1.43)	1.27 (1.12 to 1.44)	
DIMS, OR (95% CI)						
Boys	1 [Reference]	1.28 (1.16 to 1.41)	1.32 (1.20 to 1.45)	1.29 (1.17 to 1.42)	1.33 (1.20 to 1.49)	.93
Girls	1 [Reference]	1.30 (1.19 to 1.43)	1.32 (1.21 to 1.46)	1.27 (1.15 to 1.39)	1.29 (1.16 to 1.43)	
SBD, OR (95% CI)						
Boys	1 [Reference]	1.11 (1.02 to 1.21)	1.19 (1.09 to 1.30)	1.19 (1.09 to 1.30)	1.11 (1.01 to 1.22)	.40
Girls	1 [Reference]	1.26 (1.13 to 1.41)	1.31 (1.18 to 1.47)	1.24 (1.11 to 1.39)	1.21 (1.07 to 1.35)	
DA, OR (95% CI)						
Boys	1 [Reference]	1.05 (0.96 to 1.14)	1.04 (0.95 to 1.13)	1.04 (0.96 to 1.14)	1.05 (0.96 to 1.15)	.88
Girls	1 [Reference]	1.04 (0.95 to 1.13)	1.02 (0.93 to 1.11)	1.01 (0.93 to 1.11)	1.08 (0.99 to 1.19)	
SWTD, OR (95% CI)						
Boys	1 [Reference]	1.20 (1.08 to 1.32)	1.16 (1.05 to 1.29)	1.15 (1.03 to 1.27)	1.09 (0.98 to 1.22)	.14
Girls	1 [Reference]	1.13 (1.02 to 1.25)	1.25 (1.13 to 1.38)	1.07 (0.96 to 1.18)	1.01 (0.90 to 1.13)	
DOES, OR (95% CI)						
Boys	1 [Reference]	1.21 (1.10 to 1.34)	1.29 (1.17 to 1.42)	1.26 (1.14 to 1.39)	1.30 (1.17 to 1.45)	.16
Girls	1 [Reference]	1.41 (1.28 to 1.56)	1.37 (1.24 to 1.51)	1.28 (1.16 to 1.42)	1.39 (1.25 to 1.55)	
SHY, OR (95% CI)						
Boys	1 [Reference]	1.12 (1.04 to 1.20)	1.10 (1.02 to 1.18)	1.11 (1.03 to 1.19)	0.97 (0.89 to 1.05)	.15
Girls	1 [Reference]	1.09 (1.00 to 1.20)	1.13 (1.03 to 1.24)	1.01 (0.92 to 1.11)	0.89 (0.80 to 0.98)	
Shorter sleep duration, OR (95% CI)						
Boys	1 [Reference]	1.27 (1.18 to 1.37)	1.27 (1.18 to 1.38)	1.16 (1.07 to 1.25)	1.34 (1.23 to 1.46)	.42
Girls	1 [Reference]	1.20 (1.12 to 1.29)	1.25 (1.16 to 1.35)	1.13 (1.05 to 1.21)	1.40 (1.29 to 1.52)	
Longer sleep latency, OR (95% CI)						
Boys	1 [Reference]	1.21 (1.04 to 1.40)	1.08 (0.93 to 1.25)	1.06 (0.91 to 1.24)	1.35 (1.15 to 1.59)	.93
Girls	1 [Reference]	1.22 (1.05 to 1.42)	1.14 (0.97 to 1.32)	1.11 (0.95 to 1.30)	1.31 (1.11 to 1.54)	

Abbreviations: ALAN, artificial light at night; DA, disorders of arousal; DIMS, disorders of initiating and maintaining sleep; DOES, disorders of excessive somnolence; OR, odds ratio; SBD, sleep-breathing disorders; SHY, sleep hyperhidrosis; SWTD, sleep-wake transition disorders.

^a^ Includes 201 994 participants. Median ALAN exposure was 8.5 (IQR, 4.6-11.6) nanowatts/centimeters squared/steradian (nW/cm^2^/sr) for quintile 1; 19.1 (IQR, 17.6-20.8) nW/cm^2^/sr for quintile 2; 25.9 (IQR, 24.4-27.3) nW/cm^2^/sr for quintile 3; 32.4 (IQR, 30.6-34.8) nW/cm^2^/sr for quintile 4; and 47.7 (IQR, 42.0-64.0) nW/cm^2^/sr for quintile 5. Values are adjusted for sex, parental educational attainment, annual household income, district-level gross domestic product, district-level population density, and interaction item (ALAN exposure × age).

^b^ Calculated by adding an interaction item in the model.

^c^ Scores were calculated as *t* scores.

### Sensitivity Analysis

We found similar results when we additionally adjusted the main models for breastfeeding and passive smoking (eTable 3 in the Supplement). We observed similar estimates after excluding children with allergic rhinitis (eTable 4 in the Supplement) or asthma (eTable 5 in the Supplement).

## Discussion

In our cross-sectional study of 201 994 children, exposure to outdoor ALAN was associated with increased sleep scores and odds of sleep disorders, shorter sleep duration, and longer sleep latency. Furthermore, we also found that children’s age generally modified the associations of outdoor ALAN with some subtypes of sleep score and sleep disorder.

To our knowledge, this study is the first to explore the associations between outdoor ALAN exposure and SDSC-defined sleep disorders in children. Sleep consists of several dynamic dimensions. Sleep dimensions, as distinct from sleep duration or sleep pattern, are commonly used as diagnostic criteria for sleep disorders.^44^ Generally, there are many dimensions of sleep disorder in children,^25^ including sleep-wake transition disorders, DIMS, DOES, disorders of arousal, SHY, and SBD. These sleep disorder symptoms can be used to comprehensively assess sleep disorders in children through a validated tool such as the SDSC questionnaire.

Our results are consistent with those of studies from Germany and US in children and adolescents,^23,24^ but our study included more information. The prior 2 studies focused on sleep duration and sleep latency. However, in addition to short sleep duration and long sleep latency, the DIMS subtypes in the present study also reflect problems of reluctance to go to bed and difficulty falling asleep. Moreover, no studies on ALAN exposure and other sleep disorder subtypes in children have been performed. However, the association of outdoor ALAN exposure with DOES and SBD in children in this study was consistent with the results of studies among adults, which reported associations between outdoor ALAN exposure and daytime sleepiness (a symptom of DOES) and habitual snoring (a symptom of SBD).^18,19,21,22^ Our research adds to the evidence on the associations between outdoor ALAN exposure and sleep health among children, especially in low- and middle-income countries. During the past few decades, outdoor ALAN levels have increased in low- and middle-income countries to high levels of nocturnal light exposure.^17,45,46^ Effective control of outdoor ALAN might both promote children’s sleep while also benefitting other aspects of health, such as children’s mental health.^24^

We found nonlinear associations between outdoor ALAN and sleep score and sleep disorders, and the Q2 or Q3 of outdoor ALAN exposure generally had the highest β values and ORs. The mechanism underlying the nonlinear associations remains unclear, but previous studies found similar patterns when investigating associations of outdoor ALAN with weekend oversleep in US adolescents^24^ and risk of coronary heart disease in older adults.^36^ When people are highly exposed to outdoor ALAN (ie, Q4 or Q5), individual adaptive behaviors (eg, closed curtains) might attenuate the health disadvantage of outdoor ALAN, which may explain the exposure-response pattern observed in the present study. However, our results from Q4 or Q5 showed associations between outdoor ALAN exposure and children’s sleep disorders.

We found that age modified the associations of outdoor ALAN exposure with sleep score and sleep disorder, with stronger associations among children younger than 12 years. The potential mechanism for these age differences may be that early childhood is a period in which children are more sensitive to the effect of light-inhibiting melatonin and the sensitivity decreases with age.^47^ Crowley et al^48^ also found that children in early puberty were more sensitive to night light than postpubescent adolescents. However, the associations of outdoor ALAN with DOES and shorter sleep duration were stronger in children 12 years or older, which might be caused by pubertal delayed sleep phase and disrupted sleep patterns.^49^ Moreover, previous studies supported that children in middle school experience insufficient sleep time and daytime sleepiness (a symptom of DOES) due to the stress of school performance.^50^

Although a previous study^51^ found that sensitivity to light was stronger in boys than girls, we did not find a modification of sex in the associations of ALAN and sleep disorders except for the association between outdoor ALAN and SBD score, which was stronger in boys than in girls. More studies are needed to explore the role of children’s sex on the associations between outdoor ALAN exposure and sleep disorders.

### Strengths and Limitations

Our research has several strengths. The study has a large sample size with sufficient power. For example, our research found that children younger than 12 years were sensitive to outdoor ALAN exposure–associated sleep disorders. In addition, we are the first, to our knowledge, to use SDSC-defined sleep disorders to comprehensively assess children’s sleep in the study of outdoor ALAN and sleep disorders. The SDSC is a suitable tool for comprehensive assessment of sleep disorders in children. It assesses the overall status of sleep disorders and captures a number of dimensions of sleep disorders, such as DIMS.

On the other hand, our research also has limitations. First, because of the nature of the cross-sectional study design, it is not possible to establish causal relationships between outdoor ALAN and children’s sleep disorders. Because sleep disorders constitute a nonfatal chronic disorder, there may be prevalence bias in this survey of sleep disorders. Second, the light exposure in this study is outdoor ALAN based on satellite images, which is not the total light exposure, because there may also be indoor sources of ALAN or adaptive behaviors in response to outdoor sources of ALAN (eg, curtains restricting outdoor light from entering the room).^36^ Therefore, the results should be interpreted as an association between sleep disorder and outdoor ALAN rather than the total light exposure in the bedroom at night, as found in other studies on outdoor ALAN exposure and human health.^22,24,33,36^ Third, we evaluated sleep disorders on a subjective scale rather than using objective measures such as night polysomnography, which is not practical in large population-based studies. Finally, although we adjusted for several influencing factors for sleep disorder, we cannot rule out the possibility of residual confounding such as light in the room and noise.^52^

## Conclusions

The findings of this cross-sectional study suggest that there is an association between outdoor ALAN exposure and sleep disorders among children and that children’s age generally modifies these associations. These results further suggest that to reduce the severity of childhood sleep disorders, policy makers should take measures to control ALAN levels and health care professionals should develop strategies to promote healthy and adequate sleep among children.

## Notes

Supplement. **eTable 1.** Details of All Districts **eTable 2.** Distribution of Outdoor Artificial Light at Night **eTable 3.** Associations Between Quintiles of Outdoor ALAN With Sleep Disorder Score and Odds of Sleep Disorder in Children Adjusted for Additional Variables of Passive Smoking and Breastfeeding (N = 201 994) **eTable 4.** Associations Between Quintiles of Outdoor ALAN With Sleep Disorder Score and Odds of Sleep Disorder in Children Excluding Children With Allergic Rhinitis (n = 175 539) **eTable 5.** Associations Between Quintiles of Outdoor ALAN With Sleep Disorder Score and Odds of Sleep Disorder in Children Excluding Children With Asthma (n = 185 260) **eFigure 1.** Flowchart of Study Design and Participant Selection **eFigure 2.** Directed Acyclic Graph for the Association Between Outdoor Artificial Light at Night and Sleep Disorder Symptoms, Showing All Potential Covariates
