# Supplementary material for: Association Between Exposure to Outdoor Artificial Light at Night and Sleep Disorders Among Children in China
Source: JAMA Netw Open. 2022 May 20;5(5):e2213247. doi: 10.1001/jamanetworkopen.2022.13247 (PMC9123501; doi:10.1001/jamanetworkopen.2022.13247)
Supplement: Supplement. — eTable 1. Details of All Districts eTable 2. Distribution of Outdoor Artificial Light at Night eTable 3. Associations Between Quintiles of Outdoor ALAN With Sleep Disorder Score and Odds of Sleep Disorder in Children Adjusted for Additional Variables of Passive Smoking and Breastfeeding (N = 201 994) eTable 4. Associations Between Quintiles of Outdoor ALAN With Sleep Disorder Score and Odds of Sleep Disorder in Children Excluding Children With Allergic Rhinitis (n = 175 539) eTable 5. Associations Between Quintiles of Outdoor ALAN With Sleep Disorder Score and Odds of Sleep Disorder in Children Excluding Children With Asthma (n = 185 260) eFigure 1. Flowchart of Study Design and Participant Selection eFigure 2. Directed Acyclic Graph for the Association Between Outdoor Artificial Light at Night and Sleep Disorder Symptoms, Showing All Potential Covariates [file jamanetwopen-e2213247-s001.pdf]

Supplementary Online Content

Wang LB, Gong YC, Fang QL, et al. Association between exposure to outdoor artificial light at night and sleep disorders among children in China. *JAMA Netw Open*. 2022;5(5):e2213247. doi:10.1001/jamanetworkopen.2022.13247

eTable 1. Details of All Districts
eTable 2. Distribution of Outdoor Artificial Light at Night
eTable 3. Associations Between Quintiles of Outdoor ALAN With Sleep Disorder Score and Odds of Sleep Disorder in Children Adjusted for Additional Variables of Passive Smoking and Breastfeeding (N = 201 994)
eTable 4. Associations Between Quintiles of Outdoor ALAN With Sleep Disorder Score and Odds of Sleep Disorder in Children Excluding Children With Allergic Rhinitis (n = 175 539)
eTable 5. Associations Between Quintiles of Outdoor ALAN With Sleep Disorder Score and Odds of Sleep Disorder in Children Excluding Children With Asthma (n = 185 260)
eFigure 1. Flowchart of Study Design and Participant Selection
eFigure 2. Directed Acyclic Graph for the Association Between Outdoor Artificial Light at Night and Sleep Disorder Symptoms, Showing All Potential Covariates

This supplementary material has been provided by the authors to give readers additional information about their work.

© 2022 Wang LB et al. *JAMA Network Open*.

eTable 1. Details of All Districts

Province	City	Distrcit	Population (×10 000)	Area (km^2)	Population density (persons/km^2)	GDP (Yuan per capita)
Liaoning	Shenyang	Heping	70.4	58.9	11 952	99 754
Liaoning	Shenyang	Shenhe	84.4	59.5	14 185	101 141
Liaoning	Shenyang	Dadong	79.2	100.1	7912	82 692
Liaoning	Shenyang	Huanggu	94.6	66.2	14 290	46 399
Liaoning	Shenyang	Tiexi	115.9	286.1	4051	66 828
Liaoning	Shenyang	Zhanqian	30.1	82.32	3656	41 944
Liaoning	Dalian	Zhongshan	36.05	40	8990	189 618
Liaoning	Dalian	Ganjingzi	76.8	492	1911	115 893
Liaoning	Dalian	Shahekou	64.46	35	18 572	57 773
Liaoning	Dalian	Xigang	29.09	24	12 139	111 433
Liaoning	Dalian	Xishi	19.9	314.9	632	51 900
Liaoning	Fushun	Shuncheng	43	348	1236	29 019
Liaoning	Fushun	Xinfu	29.9	107.15	2790	72 631
Liaoning	Fushun	Dongzhou	30.5	604	505	68 554
Liaoning	Fushun	Wanghua	30.06	109.14	2754	24 765
Liaoning	Anshan	Tiedong	54	41.77	12 928	87 780
Liaoning	Anshan	Tiexi	37.9	29.89	12 680	92 976
Liaoning	Anshan	Lishan	40.3	48.41	8325	78 331
Liaoning	Benxi	Pingshan	33	179	1844	54 548
Liaoning	Benxi	Mingshan	41.9	413	1015	27 947
Liaoning	Benxi	Xihu	7.2	322	224	89 092
Liaoning	Dandong	Zhenxing	49.8	130.08	3828	22 262
Liaoning	Dandong	Yuanbao	21.6	91.6	2358	30 437
Liaoning	Dandong	Zhenan	19	651	292	24 567
Liaoning	Liaoyang	Baita	36.8	35	10514	40 806
Liaoning	Liaoyang	Wensheng	12.7	305	416	30 289
Liaoning	Liaoyang	Laobian	11.8	217	544	141 553
Guangdong	Guangzhou	Liwan	95.00	59.10	16 074	122 028
Guangdong	Guangzhou	Yuexiu	116.38	33.80	34 432	271 050
Guangdong	Guangzhou	Haizhu	166.31	90.40	18 397	104 499
Guangdong	Guangzhou	Tianhe	169.79	96.33	17 626	252 407
Guangdong	Guangzhou	Huangpu	109.10	484.17	2253	297 179
Guangdong	Guangzhou	Panyu	171.93	529.94	3244	114 712
Guangdong	Guangzhou	Huadu	107.55	970.04	1109	119 937
Guangdong	Guangzhou	Nansha	72.50	783.86	925	190 169
Guangdong	Guangzhou	Conghua	64.21	1974.50	325	62 312
Guangdong	Shenzhen	Luohu	102.72	78.75	13 044	210 446
Guangdong	Shenzhen	Baoan	314.9	396.61	7940	109 659
Guangdong	Shenzhen	Longgang	227.89	388.22	5870	169 869
Guangdong	Foshan	Chancheng	116.11	153.91	7544	150 082
Guangdong	Foshan	Nanhai	278.75	1071.55	2601	97 035
Guangdong	Foshan	Shunde	261.45	806	3244	116 914
Guangdong	Foshan	Gaoming	44.02	938	469	193 028
Guangdong	Foshan	Sanshui	65.34	827.69	789	177 843
Guangdong	Zhongshan	Shiqi	20.91	22.72	9203	115 804
Guangdong	Zhongshan	Dongqu	17.44	71.4	2443	153 084
Guangdong	Zhongshan	Xiaolan	33.32	147.29	2262	110 176
Guangdong	Zhongshan	Minzhong	11.31	118.87	952	72 001
Guangdong	Zhongshan	Nanlang	11.1	218.86	507	116 808
Guangdong	Zhongshan	Sanxiang	20.78	93.68	2219	87 739
Guangdong	Zhuhai	Xiangzhou	97.07	553.31	1754	150 476
Guangdong	Zhuhai	Doumeng	43.89	613.88	715	70 249
Guangdong	Maoming	Maonan	47.22	486	971	51 042
Guangdong	Maoming	Dianbai	166.64	2230	747	34 863
Xinjiang	Kashgar	Kashgar	72.23	1056.8	683	22 406

Abbreviation: GDP, gross domestic product.

© 2022 Wang LB et al. *JAMA Network Open*.

eTable 2. Distribution of Outdoor Artificial Light at Night Levels

	n	Mean (SD)	Median (IQR)	P25	P75	Minimum	Maximum
**Total**	201 994	27.85 (16.39)	25.89 (17.23)	17.58	34.81	0.02	113.48
**Province/Autonomous Region**							
Liaoning	59 754	32.96 (19.02)	29.00 (24.06)	20.19	44.25	2.33	87.60
Guangdong	131 412	23.83 (12.21)	23.59 (15.15)	15.53	30.68	0.02	113.48
Xinjiang	10 828	48.46 (21.00)	53.21 (37.22)	31.07	68.29	0.34	75.57
**Gender**							
Boys	106 378	27.72 (16.31)	25.89 (17.23)	17.58	34.81	0.02	113.48
Girls	95 616	27.99 (16.49)	25.89 (17.23)	17.60	34.83	0.02	113.48

Note: the unit of artificial light at night, nanowatts per centimeter squared per steradian ($nW/cm^2/sr$)

© 2022 Wang LB et al. *JAMA Network Open*.

eTable 3. Associations Between Quintiles of Outdoor ALAN With Sleep Disorder Score and Odds of Sleep Disorder in Children Adjusted for Additional Variables of Passive Smoking and Breastfeeding (N = 201 994)

	Q1	Q2	Q3	Q4	Q5
**β (95%CI) for outdoor ALAN**					
total sleep score [a]					
crude	Reference	0.83 (0.69, 0.98)	0.88 (0.74, 1.02)	0.74 (0.60, 0.88)	0.69 (0.53, 0.84)
adjusted [b]	Reference	0.83 (0.68, 0.98)	0.85 (0.70, 1.00)	0.65 (0.50, 0.81)	0.58 (0.41, 0.75)
DIMS score [a]					
crude	Reference	0.97 (0.83, 1.11)	0.90 (0.77, 1.04)	0.91 (0.77, 1.05)	0.96 (0.81, 1.12)
adjusted [b]	Reference	0.91 (0.76, 1.05)	0.77 (0.63, 0.92)	0.76 (0.61, 0.91)	0.87 (0.70, 1.05)
SBD score [a]					
crude	Reference	0.56 (0.42, 0.70)	0.86 (0.72, 1.00)	0.89 (0.75, 1.03)	0.84 (0.68, 1.00)
adjusted [b]	Reference	0.42 (0.28, 0.57)	0.63 (0.49, 0.78)	0.54 (0.39, 0.70)	0.37 (0.20, 0.54)
DA score [a]					
crude	Reference	0.16 (0.02, 0.30)	0.06 (−0.08, 0.20)	0.01 (−0.13, 0.15)	−0.08 (−0.23, 0.08)
adjusted [b]	Reference	0.29 (0.14, 0.43)	0.25 (0.10, 0.40)	0.19 (0.03, 0.34)	0.12 (−0.05, 0.29)
SWTD score [a]					
crude	Reference	0.47 (0.33, 0.61)	0.41 (0.27, 0.55)	0.20 (0.06, 0.34)	0.16 (0.00, 0.32)
adjusted [b]	Reference	0.42 (0.27, 0.57)	0.43 (0.29, 0.58)	0.20 (0.04, 0.35)	0.03 (−0.14, 0.20)
DOES score [a]					
crude	Reference	0.48 (0.34, 0.63)	0.68 (0.54, 0.82)	0.57 (0.43, 0.72)	0.47 (0.31, 0.63)
adjusted [b]	Reference	0.70 (0.55, 0.84)	0.79 (0.64, 0.93)	0.60 (0.44, 0.75)	0.70 (0.53, 0.87)
SHY score [a]					
crude	Reference	0.62 (0.47, 0.76)	0.55 (0.41, 0.69)	0.35 (0.21, 0.49)	0.32 (0.16, 0.48)
adjusted [b]	Reference	0.43 (0.28, 0.57)	0.48 (0.34, 0.62)	0.27 (0.12, 0.42)	−0.01 (−0.18, 0.16)
**OR (95%CI) for outdoor ALAN**					
Sleep disorder					
crude	Reference	1.23 (1.14, 1.33)	1.30 (1.21, 1.41)	1.22 (1.13, 1.32)	1.14 (1.05, 1.25)
adjusted [b]	Reference	1.34 (1.23, 1.45)	1.43 (1.32, 1.56)	1.32 (1.21, 1.44)	1.25 (1.14, 1.38)
DIMS					
crude	Reference	1.26 (1.18, 1.35)	1.29 (1.21, 1.38)	1.26 (1.18, 1.35)	1.25 (1.16, 1.35)
adjusted [b]	Reference	1.29 (1.21, 1.39)	1.32 (1.23, 1.42)	1.28 (1.19, 1.37)	1.31 (1.21, 1.43)
SBD					
crude	Reference	1.17 (1.09, 1.25)	1.26 (1.18, 1.35)	1.29 (1.21, 1.38)	1.25 (1.16, 1.34)
adjusted [b]	Reference	1.17 (1.09, 1.25)	1.24 (1.15, 1.33)	1.21 (1.12, 1.30)	1.15 (1.06, 1.24)
DA					
crude	Reference	0.97 (0.91, 1.03)	0.93 (0.87, 0.98)	0.93 (0.88, 0.99)	0.94 (0.88, 1.01)
adjusted [b]	Reference	1.04 (0.98, 1.11)	1.03 (0.97, 1.09)	1.03 (0.97, 1.10)	1.07 (1.00, 1.15)
SWTD					
crude	Reference	1.10 (1.03, 1.18)	1.12 (1.05, 1.20)	1.04 (0.97, 1.12)	1.00 (0.93, 1.08)
adjusted [b]	Reference	1.16 (1.08, 1.25)	1.21 (1.12, 1.30)	1.11 (1.03, 1.20)	1.05 (0.97, 1.15)
DOES					
crude	Reference	1.20 (1.12, 1.28)	1.23 (1.15, 1.32)	1.19 (1.11, 1.27)	1.19 (1.10, 1.28)
adjusted [b]	Reference	1.31 (1.22, 1.41)	1.33 (1.24, 1.43)	1.28 (1.18, 1.37)	1.36 (1.25, 1.47)
SHY					
crude	Reference	1.15 (1.09, 1.21)	1.09 (1.04, 1.16)	1.06 (1.00, 1.12)	1.02 (0.96, 1.09)
adjusted [b]	Reference	1.11 (1.05, 1.18)	1.11 (1.05, 1.18)	1.08 (1.01, 1.15)	0.94 (0.88, 1.01)
Shorter sleep duration					
crude	Reference	1.33 (1.27, 1.40)	1.33 (1.27, 1.40)	1.17 (1.12, 1.23)	1.51 (1.43, 1.60)
adjusted [b]	Reference	1.24 (1.17, 1.31)	1.26 (1.19, 1.34)	1.14 (1.08, 1.21)	1.37 (1.29, 1.47)
Longer sleep latency					
crude	Reference	1.19 (1.08, 1.32)	1.09 (0.98, 1.21)	1.08 (0.97, 1.20)	1.25 (1.11, 1.41)
adjusted [b]	Reference	1.22 (1.09, 1.36)	1.11 (0.99, 1.23)	1.09 (0.97, 1.22)	1.33 (1.16, 1.51)

Abbreviations: OR, odd ratio; CI, confidence interval; SWTD, sleep-wake transition disorders; DIMS, disorders of initiating and maintaining sleep; DOES, disorders of excessive somnolence; DA, disorders of arousal; SHY, sleep hyperhidrosis; SBD, sleep-breathing disorders; ALAN, artificial light at night; Q1, quintile 1; Q2, quintile 2; Q3, quintile 3; Q4, quintile 4; Q5, quintile 5.

Median: Q1, 8.5 nW/cm^2/sr; Q2, 19.1 nW/cm^2/sr; Q3, 25.9 nW/cm^2/sr; Q4, 32.4 nW/cm^2/sr; Q5, 47.7 nW/cm^2/sr.

[a] Scores were calculated as *t* scores.

[b] Adjusted for children's age, children's sex, parental education, annual household income, district-level gross domestic product, and district-level population density, passive smoking, and breastfeeding.

© 2022 Wang LB et al. *JAMA Network Open*.

eTable 4. Associations Between Quintiles of Outdoor ALAN With Sleep Disorder Score and Odds of Sleep Disorder in Children Excluding Children With Allergic Rhinitis (n = 175 539)

	Q1	Q2	Q3	Q4	Q5
**β (95%CI) for outdoor ALAN**					
total sleep score [a]					
crude	Reference	0.76 (0.61, 0.90)	0.72 (0.57, 0.87)	0.53 (0.37, 0.68)	0.56 (0.39, 0.72)
adjusted [b]	Reference	0.77 (0.61, 0.92)	0.74 (0.58, 0.90)	0.50 (0.34, 0.67)	0.52 (0.34, 0.70)
DIMS score [a]					
crude	Reference	0.90 (0.76, 1.05)	0.80 (0.65, 0.95)	0.78 (0.63, 0.93)	0.90 (0.73, 1.06)
adjusted [b]	Reference	0.86 (0.71, 1.02)	0.70 (0.54, 0.85)	0.67 (0.50, 0.83)	0.87 (0.69, 1.05)
SBD score [a]					
crude	Reference	0.41 (0.27, 0.55)	0.63 (0.49, 0.77)	0.51 (0.37, 0.66)	0.58 (0.43, 0.74)
adjusted [b]	Reference	0.32 (0.17, 0.46)	0.51 (0.36, 0.65)	0.34 (0.18, 0.49)	0.28 (0.11, 0.45)
DA score [a]					
crude	Reference	0.15 (0.00, 0.30)	0.04 (−0.11, 0.19)	−0.10 (−0.25, 0.06)	−0.10 (−0.26, 0.07)
adjusted [b]	Reference	0.29 (0.13, 0.45)	0.24 (0.09, 0.40)	0.09 (−0.07, 0.26)	0.12 (−0.06, 0.30)
SWTD score [a]					
crude	Reference	0.44 (0.29, 0.59)	0.32 (0.17, 0.47)	0.04 (−0.11, 0.19)	0.09 (−0.08, 0.26)
adjusted [b]	Reference	0.38 (0.23, 0.54)	0.35 (0.19, 0.51)	0.07 (−0.10, 0.23)	−0.03 (−0.22, 0.15)
DOES score [a]					
crude	Reference	0.44 (0.29, 0.59)	0.59 (0.44, 0.74)	0.52 (0.36, 0.67)	0.39 (0.22, 0.55)
adjusted [b]	Reference	0.67 (0.51, 0.82)	0.72 (0.57, 0.88)	0.57 (0.41, 0.73)	0.68 (0.50, 0.86)
SHY score [a]					
crude	Reference	0.55 (0.41, 0.70)	0.43 (0.28, 0.57)	0.17 (0.01, 0.32)	0.24 (0.07, 0.40)
adjusted [b]	Reference	0.37 (0.22, 0.52)	0.38 (0.23, 0.53)	0.16 (0.00, 0.32)	−0.07 (−0.24, 0.11)
**OR (95%CI) for outdoor ALAN**					
Sleep disorder					
crude	Reference	1.21 (1.11, 1.32)	1.24 (1.14, 1.35)	1.12 (1.03, 1.23)	1.08 (0.98, 1.19)
adjusted [b]	Reference	1.32 (1.21, 1.45)	1.39 (1.27, 1.52)	1.24 (1.13, 1.37)	1.23 (1.10, 1.37)
DIMS					
crude	Reference	1.23 (1.14, 1.32)	1.25 (1.16, 1.35)	1.20 (1.12, 1.30)	1.22 (1.13, 1.33)
adjusted [b]	Reference	1.27 (1.17, 1.37)	1.29 (1.20, 1.39)	1.23 (1.14, 1.33)	1.31 (1.19, 1.44)
SBD					
crude	Reference	1.14 (1.05, 1.24)	1.22 (1.13, 1.32)	1.19 (1.10, 1.29)	1.17 (1.08, 1.28)
adjusted [b]	Reference	1.16 (1.06, 1.26)	1.24 (1.14, 1.35)	1.18 (1.08, 1.29)	1.14 (1.04, 1.25)
DA					
crude	Reference	0.96 (0.90, 1.03)	0.92 (0.87, 0.99)	0.91 (0.85, 0.97)	0.93 (0.87, 1.00)
adjusted [b]	Reference	1.04 (0.97, 1.11)	1.03 (0.97, 1.11)	1.02 (0.95, 1.09)	1.07 (0.99, 1.16)
SWTD					
crude	Reference	1.13 (1.05, 1.22)	1.11 (1.03, 1.20)	1.02 (0.94, 1.10)	1.00 (0.91, 1.08)
adjusted [b]	Reference	1.19 (1.10, 1.29)	1.20 (1.11, 1.31)	1.09 (1.00, 1.19)	1.06 (0.96, 1.16)
DOES					
crude	Reference	1.17 (1.09, 1.26)	1.21 (1.12, 1.30)	1.17 (1.08, 1.26)	1.16 (1.07, 1.26)
adjusted [b]	Reference	1.30 (1.20, 1.40)	1.32 (1.22, 1.43)	1.27 (1.17, 1.37)	1.36 (1.24, 1.49)
SHY					
crude	Reference	1.13 (1.06, 1.20)	1.04 (0.98, 1.10)	1.00 (0.94, 1.06)	1.01 (0.94, 1.08)
adjusted [b]	Reference	1.08 (1.01, 1.15)	1.06 (0.99, 1.13)	1.03 (0.96, 1.10)	0.92 (0.85, 0.99)
Shorter sleep duration					
crude	Reference	1.34 (1.27, 1.41)	1.31 (1.25, 1.38)	1.18 (1.12, 1.25)	1.51 (1.43, 1.61)
adjusted [b]	Reference	1.23 (1.16, 1.30)	1.23 (1.16, 1.31)	1.14 (1.07, 1.21)	1.38 (1.28, 1.48)
Longer sleep latency					
crude	Reference	1.19 (1.06, 1.33)	1.07 (0.96, 1.20)	1.07 (0.95, 1.20)	1.26 (1.11, 1.43)
adjusted [b]	Reference	1.21 (1.08, 1.36)	1.09 (0.97, 1.24)	1.09 (0.96, 1.23)	1.35 (1.18, 1.56)

Abbreviations: OR, odd ratio; CI, confidence interval; SWTD, sleep-wake transition disorders; DIMS, disorders of initiating and maintaining sleep; DOES, disorders of excessive somnolence; DA, disorders of arousal; SHY, sleep hyperhidrosis; SBD, sleep-breathing disorders; ALAN, artificial light at night; Q1, quintile 1; Q2, quintile 2; Q3, quintile 3; Q4, quintile 4; Q5, quintile 5.

Median: Q1, 8.5 nW/cm^2/sr; Q2, 19.1 nW/cm^2/sr; Q3, 25.9 nW/cm^2/sr; Q4, 32.4 nW/cm^2/sr; Q5, 47.7 nW/cm^2/sr.

[a] Scores were calculated as *t* scores.

[b] Adjusted for children's age, children's sex, parental education, annual household income, district-level gross domestic product, and district-level population density.

© 2022 Wang LB et al. *JAMA Network Open*.

eTable 5. Associations Between Quintiles of Outdoor ALAN With Sleep Disorder Score and Odds of Sleep Disorder in Children Excluding Children With Asthma (n = 185 260)

	Q1	Q2	Q3	Q4	Q5
**β (95%CI) for outdoor ALAN**					
total sleep score [a]					
crude	Reference	0.79 (0.65, 0.94)	0.80 (0.66, 0.94)	0.66 (0.51, 0.81)	0.60 (0.44, 0.76)
adjusted [b]	Reference	0.77 (0.62, 0.92)	0.75 (0.60, 0.91)	0.55 (0.39, 0.71)	0.46 (0.28, 0.63)
DIMS score [a]					
crude	Reference	0.92 (0.77, 1.06)	0.82 (0.68, 0.96)	0.86 (0.71, 1.00)	0.90 (0.74, 1.06)
adjusted [b]	Reference	0.88 (0.73, 1.03)	0.70 (0.55, 0.85)	0.71 (0.55, 0.87)	0.82 (0.64, 1.00)
SBD score [a]					
crude	Reference	0.53 (0.39, 0.67)	0.77 (0.63, 0.91)	0.75 (0.60, 0.89)	0.73 (0.57, 0.89)
adjusted [b]	Reference	0.38 (0.23, 0.53)	0.55 (0.40, 0.70)	0.42 (0.27, 0.58)	0.27 (0.09, 0.44)
DA score [a]					
crude	Reference	0.11 (-0.04, 0.26)	0.02 (−0.12, 0.17)	−0.03 (−0.18, 0.12)	−0.11 (−0.27, 0.05)
adjusted [b]	Reference	0.20 (0.05, 0.35)	0.17 (0.02, 0.32)	0.10 (-0.06, 0.26)	0.04 (−0.14, 0.21)
SWTD score [a]					
crude	Reference	0.46 (0.32, 0.61)	0.37 (0.23, 0.52)	0.16 (0.01, 0.31)	0.11 (−0.05, 0.27)
adjusted [b]	Reference	0.38 (0.23, 0.53)	0.36 (0.21, 0.52)	0.12 (−0.03, 0.28)	−0.08 (−0.26, 0.10)
DOES score [a]					
crude	Reference	0.45 (0.31, 0.60)	0.64 (0.49, 0.79)	0.51 (0.36, 0.66)	0.40 (0.24, 0.57)
adjusted [b]	Reference	0.67 (0.51, 0.82)	0.73 (0.58, 0.88)	0.52 (0.36, 0.68)	0.61 (0.44, 0.79)
SHY score [a]					
crude	Reference	0.60 (0.45, 0.74)	0.53 (0.39, 0.67)	0.31 (0.17, 0.46)	0.28 (0.12, 0.44)
adjusted [b]	Reference	0.38 (0.23, 0.53)	0.43 (0.28, 0.58)	0.21 (0.06, 0.36)	−0.08 (−0.26, 0.09)
**OR (95%CI) for outdoor ALAN**					
Sleep disorder					
crude	Reference	1.21 (1.11, 1.31)	1.27 (1.17, 1.38)	1.18 (1.09, 1.29)	1.10 (1.00, 1.21)
adjusted [b]	Reference	1.31 (1.20, 1.43)	1.40 (1.28, 1.53)	1.28 (1.17, 1.41)	1.22 (1.10, 1.35)
DIMS					
crude	Reference	1.24 (1.16, 1.33)	1.25 (1.17, 1.34)	1.23 (1.14, 1.32)	1.23 (1.13, 1.33)
adjusted [b]	Reference	1.28 (1.19, 1.38)	1.29 (1.20, 1.39)	1.25 (1.16, 1.35)	1.29 (1.18, 1.41)
SBD					
crude	Reference	1.16 (1.08, 1.25)	1.26 (1.18, 1.36)	1.24 (1.15, 1.33)	1.22 (1.13, 1.32)
adjusted [b]	Reference	1.16 (1.07, 1.25)	1.24 (1.15, 1.34)	1.17 (1.08, 1.26)	1.12 (1.03, 1.23)
DA					
crude	Reference	0.94 (0.88, 1.00)	0.92 (0.86, 0.98)	0.92 (0.86, 0.98)	0.92 (0.86, 0.99)
adjusted [b]	Reference	1.00 (0.94, 1.07)	1.00 (0.94, 1.07)	1.00 (0.94, 1.08)	1.04 (0.96, 1.12)
SWTD					
crude	Reference	1.09 (1.01, 1.17)	1.11 (1.03, 1.20)	1.03 (0.96, 1.11)	0.96 (0.88, 1.04)
adjusted [b]	Reference	1.15 (1.06, 1.24)	1.19 (1.10, 1.29)	1.09 (1.01, 1.19)	1.00 (0.92, 1.10)
DOES					
crude	Reference	1.19 (1.11, 1.28)	1.22 (1.13, 1.31)	1.16 (1.08, 1.25)	1.16 (1.07, 1.26)
adjusted [b]	Reference	1.31 (1.21, 1.41)	1.32 (1.22, 1.42)	1.25 (1.15, 1.35)	1.33 (1.21, 1.45)
SHY					
crude	Reference	1.14 (1.08, 1.21)	1.08 (1.02, 1.15)	1.06 (0.99, 1.12)	1.02 (0.95, 1.09)
adjusted [b]	Reference	1.09 (1.03, 1.16)	1.09 (1.03, 1.16)	1.06 (1.00, 1.14)	0.92 (0.85, 0.99)
Shorter sleep duration					
crude	Reference	1.32 (1.25, 1.38)	1.30 (1.24, 1.37)	1.17 (1.11, 1.23)	1.49 (1.41, 1.58)
adjusted [b]	Reference	1.24 (1.17, 1.31)	1.25 (1.17, 1.32)	1.14 (1.08, 1.21)	1.36 (1.27, 1.46)
Longer sleep latency					
crude	Reference	1.21 (1.09, 1.35)	1.11 (0.99, 1.24)	1.10 (0.98, 1.23)	1.27 (1.12, 1.44)
adjusted [b]	Reference	1.25 (1.11, 1.40)	1.14 (1.01, 1.28)	1.11 (0.99, 1.25)	1.37 (1.20, 1.58)

Abbreviations: OR, odd ratio; CI, confidence interval; SWTD, sleep-wake transition disorders; DIMS, disorders of initiating and maintaining sleep; DOES, disorders of excessive somnolence; DA, disorders of arousal; SHY, sleep hyperhidrosis; SBD, sleep-breathing disorders; ALAN, artificial light at night; Q1, quintile 1; Q2, quintile 2; Q3, quintile 3; Q4, quintile 4; Q5, quintile 5.

Median: Q1, 8.5 nW/cm^2/sr; Q2, 19.1 nW/cm^2/sr; Q3, 25.9 nW/cm^2/sr; Q4, 32.4 nW/cm^2/sr; Q5, 47.7 nW/cm^2/sr.

[a] Scores were calculated as *t* scores..

[b] Adjusted for children's age, children's gender, parental education, annual household income, district-level gross domestic product, and district-level population density.sex

© 2022 Wang LB et al. *JAMA Network Open*.

eFigure 1. Flowchart of Study Design and Participant Selection

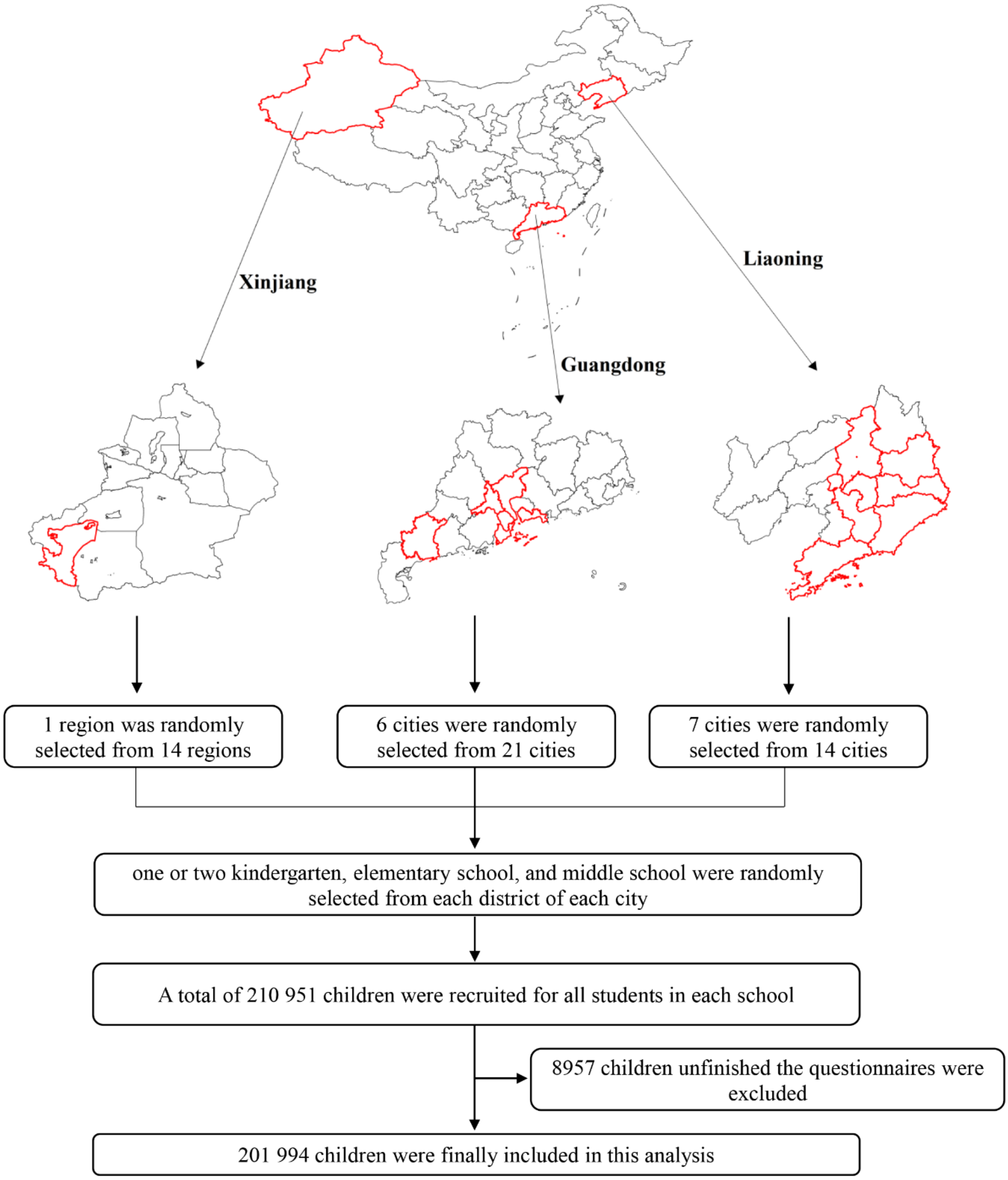

© 2022 Wang LB et al. *JAMA Network Open*.

eFigure 2. Directed Acyclic Graph for the Association Between Outdoor Artificial Light at Night and Sleep Disorder Symptoms, Showing All Potential Covariates

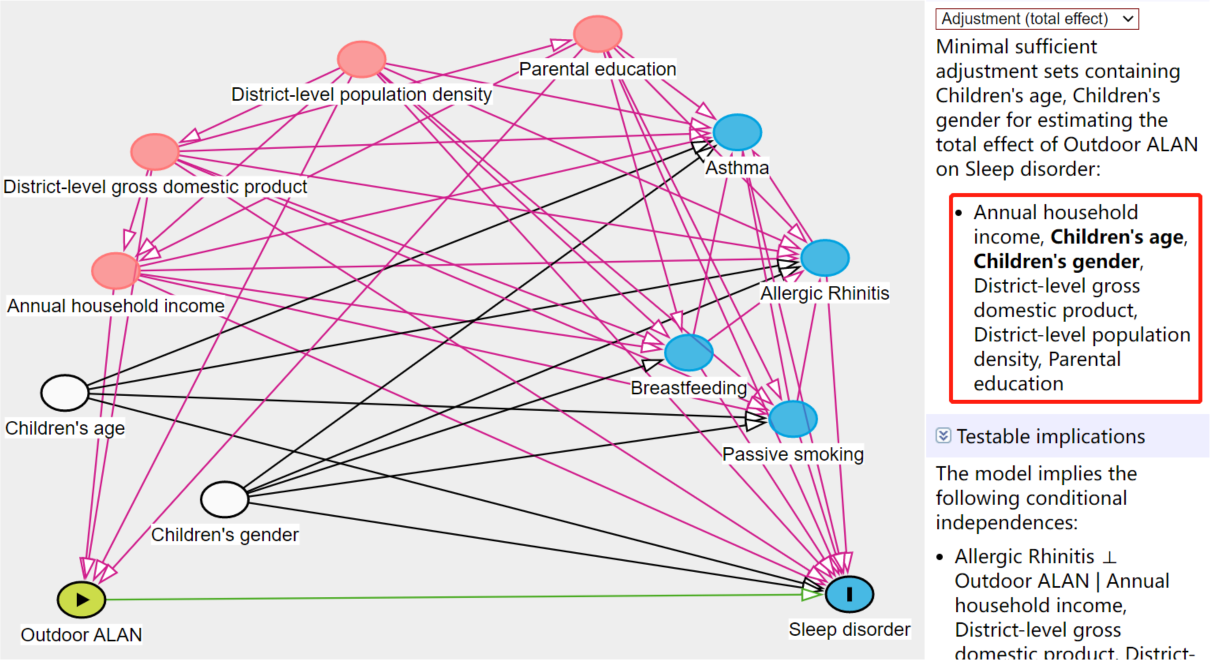

© 2022 Wang LB et al. *JAMA Network Open*.
